# Exploring Patient Activation and Compliance in Patients with Different Rheumatological Disorders

**DOI:** 10.3390/healthcare13010071

**Published:** 2025-01-02

**Authors:** Haya M. Almalag, Nora Alosaimi, Reem Alqahtani, Rahaf Alharbi, Abdulrahman S. Alarfaj, Mohammed A. Omair, Mohamed Bedaiwi, Iman Qurtas, Ibrahim Almaghlouth, Jawza F. Alsabhan, Bashayr Alsuwayni, Lobna Al Juffali

**Affiliations:** 1 Department of Clinical Pharmacy, College of Pharmacy, King Saud University, Riyadh 11451, Saudi Arabia; nalosaimi1@ksu.edu.sa (N.A.); reem.alqqahtani@gmail.com (R.A.); rahaff.alharbi0@gmail.com (R.A.); laljaffali@ksu.edu.sa (L.A.J.); 2 Rheumatology Unit, Department of Medicine, King Saud University, Riyadh 11451, Saudi Arabia; asarfaj@ksu.edu.sa (A.S.A.); momair@ksu.edu.sa (M.A.O.); mbedaiwi@ksu.edu.sa (M.B.); ialmaghlouth@gmail.com (I.A.); 3 College of Medicine Research Centre, College of Medicine, King Saud University, Riyadh 11451, Saudi Arabia; 4 Department of Pharmaceutical Services, King Saud University and Medical City, Riyadh 11451, Saudi Arabia

**Keywords:** rheumatic diseases, patient participation, compliance

## Abstract

**Purpose:** This study aimed to assess patient activation using patient activation measure 13 (PAM-13) in systemic lupus erythematosus (SLE), psoriatic arthritis (PsA), and axial spondyloarthritis (axSPA). **Patients and methods:** A cross-sectional study was conducted involving patients with three rheumatological conditions (SLE, PsA, and axSPA). Patients were contacted either at the clinic or through social media platforms. Data, including demographics, PAM 13, Arabic compliance questionnaire for rheumatology (ACQR), and disease-related activity scores, were collected electronically. The analyses included Chi-squared tests, linear regression, and binary logistic regression. **Results:** Overall, 418 patients were recruited (SLE = 323, PsA = 65, and axSPA = 30), with a mean (±SD) age of 42 ± 11 years and a female predominance (88%). PAM-13 scores did not significantly differ between the rheumatological disorders. Patients with axSPA showed significantly higher compliance than those with SLE or PsA (*p* = 0.012). In regression models, patients with PsA were more likely to be in activation level 1, with an OR of 2.890 (95% CI: 1.044–8.000, *p* = 0.0041), whereas patients with axSPA were more likely to be in activation level 4, with an OR of 2.460 (95% CI: 1.122–5.393, *p* = 0.025). The SLEDAI score was inversely related to the PAM-13 score (Pearson’s correlation coefficient = −0.221, *p* < 0.001). **Conclusions:** This study explored the levels of activation and medication compliance in different rheumatological conditions. Larger studies are needed to confirm these findings and explore the challenges and opportunities for improving compliance and activation.

## 1. Introduction

In chronic diseases, as in other illnesses, patient-centered care requires a shift in the practice and design of the healthcare system [1]. Patients should be considered active members of the healthcare team to ensure consistency in delivering high-quality healthcare services that can translate into positive patient-related outcomes [2]. The World Health Organization (WHO) started an initiative in 2021 called Patients for Patient Safety, which focuses on engaging and empowering patients and bringing their voices to the healthcare system as part of the WHO’s global strategies for people-centered and integrated health services, an essential element for quality universal health coverage [3]. Patient activation is a mediator of patient-centered care according to the structure–process–outcome model for improving healthcare quality [2].

Given the complex and chronic nature of rheumatic disorders, patient activation improves patient-related outcomes [4]. In chronic illnesses, patient activation affects patient behavior, such as compliance, and disease outcome, which is evident in terms of activity [5]. Patients with higher activation levels experience significantly better health outcomes and lower rates of doctor office and emergency room visits [6].

Patient activation was assessed using the patient activation measure (PAM-13), using 10 items [6,7]. Since its development by Hibbard in 2004, it has been a fundamental variable in over 800 research publications globally [8]. Assessing baseline patient activation in chronic diseases aims to tailor supportive measures, increase activation, and achieve positive long-term disease-related outcomes [9,10,11]. With the current increased prevalence of rheumatic illnesses and their projected rise, these conditions pose a significant burden on patients and the economy [12]. The 2021 American College of Rheumatology guidelines emphasize that clinician and patient decision making should be supported, highlighting the importance of patient engagement in rheumatological disorders [13]. Rheumatoid arthritis (RA) is a well-studied joint disease, but other rheumatological disorders receive less attention regarding patient behavior (i.e., activation) and related outcomes.

To our knowledge, no study has assessed patient activation in axial spondylarthritis or psoriatic arthritis. Wang et al. reported on patient activation in Chinese patients with SLE, but evidence remains scarce [14]. This study aimed to evaluate patient activation and compliance using Arabic PAM-13 and the Arabic compliance questionnaire for rheumatology (ACQR) in patients with rheumatologic disorders other than RA, as our team previously explored patient activation and compliance in RA on both single- and multicenter levels [4,5,15,16]. The reason is that these disorders are the most studied in the literature in Saudi Arabia (following RA). It is reported that the prevalence of SLE is estimated to be the highest following RA [17]. This is followed by psoriatic arthritis, in which 10% of patients of the projected 5.3% with psoriasis will develop arthritis [18,19]. Finally, many studies exploring axial spondylarthritis have reported its profile [20,21,22]. In addition, due to the impact of these disorders on the population, specialized rheumatology clinics for these disorders in particular have been established at our main study site (King Saud University Medical City) [15].

## 2. Material and Methods

### 2.1. Study Design and Setting

This cross-sectional survey was conducted on consenting patients, capturing patient activation in individuals with confirmed diagnoses according to international standardized diagnostic criteria. Systemic lupus erythematosus was diagnosed based on the 2019 European League Against Rheumatism/American College of Rheumatology Classification Criteria [23]. Axial spondyloarthritis and psoriatic arthritis were diagnosed according to the Spondyloarthropathy Study Group (ESSG) criteria and the Classification of Psoriatic Arthritis (CASPAR), respectively [24]. This study was observational and aligned with the Strengthening the Reporting of Observational Studies in Epidemiology (STROBE) guidelines [25]. Patients were recruited from two major sites: specialized rheumatology outpatient clinics at King Saud University Medical City and the Saudi Society of Rheumatology Charitable Organisation (Charitable Association for Rheumatic Diseases), both located in Riyadh, Saudi Arabia.

### 2.2. Participants and Recruitment

Adult patients (>18 years) with a confirmed diagnosis of one of the disorders examined in this study (i.e., systemic lupus erythematosus (SLE), psoriatic arthritis (PsA), and axial spondyloarthritis (axSPA)) attending specialized rheumatology clinics at KSUMC were recruited. Each time slot of a specialized clinic included a group of patients with a specific disorder [26]. The research team approached patients in the waiting area to invite them to participate, either in person (the number of verbally consenting patients was obtained) or through the charitable association’s phone directory. After a complete explanation of the study, electronic consent with a survey form was sent via social media platforms, mainly WhatsApp^©^ (Acton and Koum, 2009, Mountain View, CA, USA), or using the research team’s iPad^©^ for the form to be filled out in the clinic.

### 2.3. Variables and Data Source

An electronic Google^©^ (Google LLC, 2008, Menlo Park, CA, USA) Form was used in this study. The form was structured into multiple parts: study information sheet, consent form, sociodemographic characteristics, other comorbidities, medication, disease-related questions, Arabic-translated and validated PAM-13 [7], Arabic-translated and validated compliance questionnaire for rheumatology (CQR) [26,27], and disease activity scores (Systemic Lupus Erythematosus Disease Activity Index (SLEDAI) [28], validated Arabic Bath Ankylosing Spondylitis Functional Index (BASFI) [29], and validated Arabic Psoriatic Arthritis Impact of Disease (PsAID) questionnaire [30]).

### 2.4. Measurements

#### 2.4.1. PAM-13

Developed by Hibbard et al. in 2004 [6], this survey initially had 22 items but was reduced to 13 [7]. The survey is under license of use by Insignia Health. It is available in multiple languages, including Arabic. The survey evaluates the patients’ involvement in managing their health by answering 13 questions on a 5-item Likert scale. Responses are uploaded to the Insignia website, where the total score and activation level are computed. Patients fall into one of the following four activation levels: PAM1 (disengaged and overwhelmed), PAM2 (becoming aware but still struggling), PAM3 (taking action and gaining control), and PAM4 (maintaining behaviors and pushing further). Higher levels indicate more active patients.

#### 2.4.2. Compliance Measure

The CQR is a validated survey developed by Hughes et al. [27] to assess compliance among patients with rheumatological conditions. The scale was translated and validated in Arabic [26]. It contains five items in which the patient evaluates compliance by rating statements from 0 to 4. The score is then computed using the equation provided by the developer, and patients are classified as having low or high compliance.

### 2.5. Disease Activity Markers

#### 2.5.1. SLEDAI

The SLEDAI is a well-known and publicly available score that assesses disease activity in patients with SLE based on 24 items with different clinical and laboratory markers [28]. It classifies disease activity into 27 levels.

#### 2.5.2. BASFI

The BAFSI uses a scale of 0–10 covering the five major functional disability symptoms associated with AS [31]. It comprises six questions translated and validated in Arabic [29].

#### 2.5.3. PsAID

Developed and validated by the European Alliance of Associations for Rheumatology (EULAR) [32], the PsAID has 12 items, which include patient scores on the effect of PsA on work, functional capacity, and sleep on a scale of 1–10. The survey was recently translated and validated in the Arabic language [33].

### 2.6. Bias, Study Size, and Statistical Methods

All participants with a confirmed diagnosis of SRC were approached and invited. Additionally, the names and contact information of consenting patients provided by the charitable association were obtained to eliminate selection bias. Insignia Health determined that the sample size needed for PAM-13 applicability should not exceed 75. After collection, the data were exported from Google Forms, coded, and entered into the Statistical Package for Social Sciences (SPSS) version 28 (IBM Corp., Armonk, NY, USA) [34]. Descriptive data were reported as means and standard deviations or medians, and interquartile ranges were reported according to the normality of distribution. Categorical data were reported as numbers and percentages. Analysis of Variance (ANOVA) was used to compare the means of normally distributed data, and the Kruskal–Wallis test was used for non-normally distributed data. For categorical variables, the Chi-square test was used. Binary logistic and linear regressions were performed as appropriate to predict changes in PAM as continuous or categorical variables, considering confounders.

### 2.7. Ethical Consecrations and Permissions

All participants provided electronic consent for inclusion in the study. Patient identifiers were not reported to ensure anonymity or confidentiality. The Ethics Committee of King Saud University and Medical City (Institutional Review Board project number E-23-7799) approved this study. A PAM-13 license was obtained (license number: 1570198456-1601820856). Permission to use these tools was obtained from all authors.

## 3. Results

### 3.1. Demographics

Overall, 418 patients were recruited (SLE = 323, PsA = 65, and axSPA = 30) with a mean (standard deviation [SD]) age of 42 (11) years and 88% being female. Participants were mainly unemployed (58%), lived with family (96%), were non-smokers (93%), had other comorbidities (56%), and had a median (interquartile range) disease duration of 11 (11–13) years. The participants used conventional disease-modifying agents (cDMARDs, 93%), and 26% used biological or targeted synthetic DMARDs. In addition, approximately one-third of the patients (38%) used corticosteroids. In the univariate analysis, participants differed between rheumatological disorders in age, sex, employment, smoking, disease duration, the presence of other comorbidities, and the use of DMARD therapy. The highest age was observed in the AS group. The SLE group had the highest proportion of female patients, unemployment, being a non-smoker, other comorbidities, and DMARDs use (Table 1).

### 3.2. Disease Activity Scores

Patients with SLE were quiescent or had mild disease activity (61% and 24%, respectively). Patients with PsA had moderate disease activity based on the PsAID score (37%) and active disease in BASDAI (73%) (Table 1).

### 3.3. PAM-13

**Figure 1:**
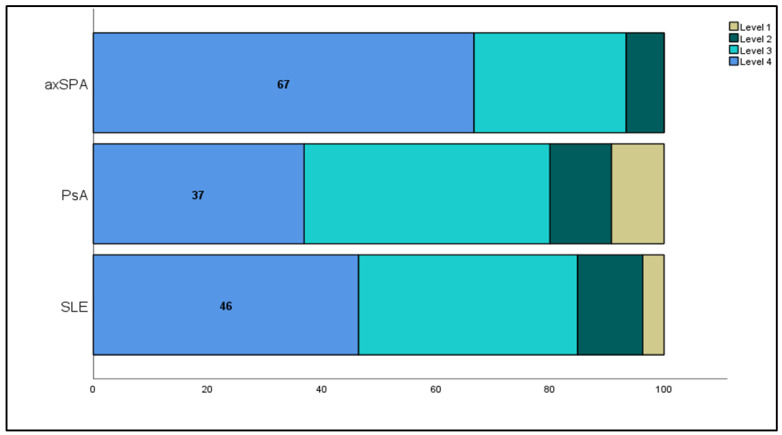
Patient activation levels stratified by disease. PAM1: disengaged and overwhelmed; PAM2: becoming aware but still struggling; PAM3: taking action and gaining control; PAM4: maintaining behaviors and pushing further. SLE: systemic lupus erythematosus; PsA: psoriatic arthritis; axSPA: axial spondylarthritis; PAM: patient activation measure.

**Figure 2:**
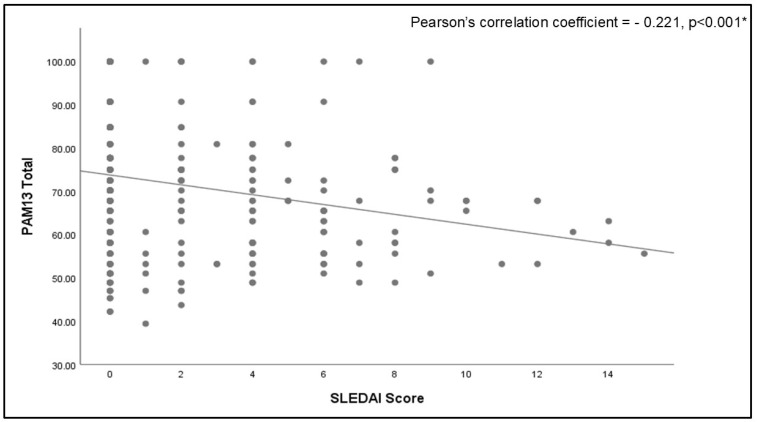
Correlation between total Patient Activation Measure 13 score and SLEDAI score. * Significant at a level of *p* < 0.05; PAM: patient activation measure; SLEDAI: Systemic Lupus Erythematosus Disease Activity Index.

**Figure 3:**
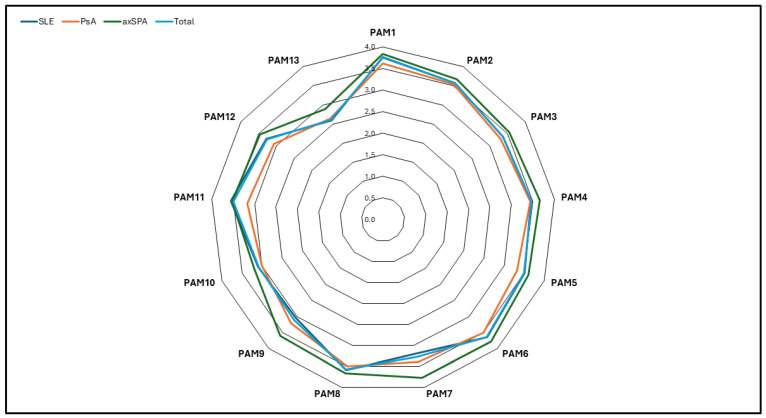
Elements of PAM-13 and different rheumatological conditions. For complete survey items, please refer to Insignia Health (https://www.insigniahealth.com/pam/ accessed on 1 January 2023). SLE: systemic lupus erythematosus; PsA: psoriatic arthritis; axSPA: axial spondylarthritis; PAM: patient activation measure.

Patient activation measures were not significantly different between rheumatological disorders; however, there was a trend of higher activation in axSPA than in other disorders (Figure 1). The SLEDAI was inversely related to the PAM-13 score, with a weak but significant Pearson’s correlation coefficient of −0.221 (95% confidence interval (CI): −0.323 to −0.115, *p* < 0.001) (Figure 2). The other disease activity scores assessed for PsA and axSPA were unrelated to patient activation (Supplementary Figures S1 and S2). With the use of a radar chart, it was observed that the PAM-13 items were very similar across different rheumatological disorders compared to the total. Minor elevations in PAM scores for items 7, 8, 9, and 13 were observed in patients with axSPA, which could explain the slightly higher activation. Figure 3 shows the lowest total patient activation score in PAM item 13, representing confidence in maintaining lifestyle changes, and the highest score in PAM item 6, reflecting confidence in healthcare providers.

### 3.4. CQR

**Figure 4:**
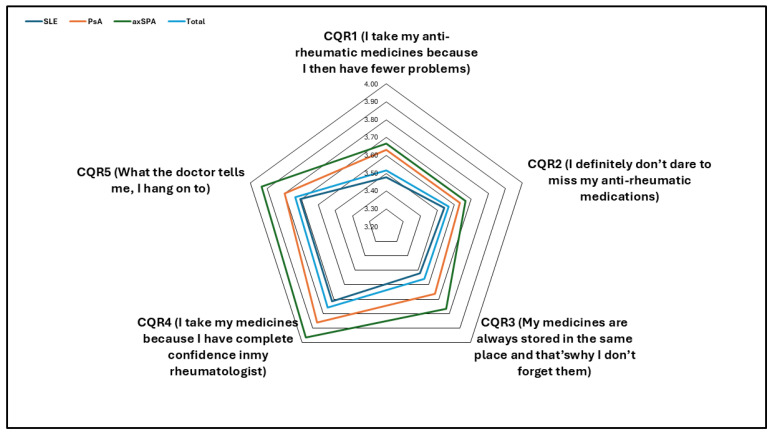
Elements of compliance questionnaire for rheumatology and different rheumatological conditions. SLE: systemic lupus erythematosus; PsA: psoriatic arthritis; axSPA: axial spondylarthritis; CQR: compliance questionnaire for rheumatology.

Compliance was categorized as low or high, with most participants having high compliance (85%), which did not differ between reported rheumatological conditions (*p* = 0.894). When reported as a continuous score, participants with axSPA showed significantly higher compliance than those with SLE or PsA (*p* = 0.012). In addition, the visualization of CQR items using a radar chart indicated that higher compliance in patients with axSPA was related to higher scores in the following items: CQR 3 (storing items in the same place), CQR 4 (confidence in the rheumatologist), and CQR 5 (complying with doctors’ instructions). The lowest score was for the belief that taking antirheumatic medications may lead to fewer problems (Figure 4). No correlation was observed between the disease activity scores and compliance.

### 3.5. Regression Analysis

Activation levels were assessed using a binary logistic regression model, reporting the odds ratio for activation levels 1, 2, 3, or 4, which were approximately the same in the SLE group. Patients with PsA were most likely to be at activation level 1 with an OR of 2.890 (95% CI of 1.044–8.000, *p* = 0.0041). Conversely, patients with axSPA were most likely to be at activation level 4 with an OR of 2.460 (95% CI: 1.122–5.393, *p* = 0.025). In using linear regression to predict activation in patients with rheumatological disorders, patients with PsA had a lower activation value with a beta of −4.272 (95% CI: −8.480–0.063, *p* = 0.047) compared to patients without PsA. Binary logistic regression was used to predict the odds of compliance with no disorder, which showed significantly higher odds than others. However, in using linear regression, compliance was lower in patients with SLE, with a beta of −0.750 (95% CI: −1.264 to −0.236, *p* = 0.004), and higher in patients with axSPA, with a beta of 0.930 (95% CI: 0.093–1.768, *p* = 0.030) (Table 2).

## 4. Discussion

This study investigated activation in patients with SLE, PsA, and axSPA in Saudi Arabia. Patient activation was not significantly different among the three conditions examined in this study. However, a direct comparison between these three disorders may be compromised by the differing characteristics of each disorder. The relationship between decision making, medication-taking behavior, and activation in chronic conditions is complex [35]. Owing to a lack of data on axSPA and PsA activation, comparisons were not possible. Wang et al. [14] reported that multiple factors, such as health literacy, quality of healthcare, and social support, affect activation in patients with SLE. All these factors also contribute to activation in other rheumatic diseases; therefore, no deviation was observed between disorders. Other factors contributing to activation in relation to each rheumatic disorder should be addressed separately in future qualitative studies. The significant association between high patient activation in patients with SLE and decreased disease activity can be directly related to patient compliance, as demonstrated by the low ACQR in our results. This finding underscores the importance of patient engagement, empowerment, and activation in influencing patient behavior and disease outcomes.

In general, our finding is consistent with that of Fortin et al. [36], who stated that positive self-management behaviors can reduce disease flare-ups and organ damage. High compliance may be indicative of patients’ knowledge and confidence in managing their disease. However, the level of patient activation was not significantly associated with changes in disease activity of either axSPA or PsA. This finding may be attributed to the small number of patients in these groups.

Patient activation improves health outcomes [37,38]. According to PAM-13, patient activation can be classified into four levels, where level 1 is low, and level 4 is high. Most patients with axSPA were at level 4, indicating that they had the knowledge and confidence to self-manage their condition. In our study, patients with axSPA were younger and had fewer comorbidities than those with SLE or PsA. High patient activation is often associated with young age, whereas low patient activation is linked with older adults and comorbidities [39,40]. Patients with low disease activity may require assistance from healthcare professionals to improve their knowledge, skills, and confidence in dealing with their diseases. These findings on disease activation levels can guide future research on implementing interventions to improve health outcomes. Patient activation supports patient-centered care in two ways. First, patients with high activation levels can play a role in adhering to health-promoting behaviors, thus becoming active members of the healthcare team and enhancing their health outcomes. Second, understanding the patients in every institution allows for the implementation and testing of specific interventions to improve patient activation and, consequently, patient outcomes.

To our knowledge, this is the first study in Saudi Arabia to provide essential data on patient engagement across three rheumatological conditions. Patient activation was assessed using 13 indirect measures that relied on participants’ self-reports, which may introduce biases such as social desirability bias. The cross-sectional design of this study presents limitations, as the measurements were performed simultaneously. The results of this study are not generalizable, and further studies are required. Older patients and those with comorbidities were less likely to participate, highlighting a limitation in the study population. Exploring the factors affecting patient activation is valuable for understanding the relationship between patient activation and disease activity. Qualitative studies are needed to understand the factors affecting patient activation, as they are challenging to explore in observational studies.

## 5. Conclusions

Understanding patient behavior and how it affects outcomes is crucial in chronic and inflammatory conditions. Although not significant, patients had different activation levels based on their rheumatological conditions, which could be related to unknown underlying factors. Compliance differed across patient groups. Therefore, patients with low disease compliance should be subject to interventions, further activation, and education. This study provides baseline data to which enhancement measures can be applied in clinical settings to design a holistic approach for patient-centered care.

## Figures and Tables

**Table 1:** Demographic characteristics of different participants with rheumatological disorders.

		Total (n = 418)	SLE (n = 323)	PsA (n = 65)	axSPA (n = 30)	*p* Value
Sex, N (%)	Male	52 (12.4)	21 (40.4)	19 (36.5)	12 (23.1)	<0.001 *
	Female	366 (87.6)	302 (82.5)	46 (12.6)	18 (4.9)	
Age, years, mean (SD)		42 (11)	42 (11)	42 (10)	43 (9)	0.006 *
Education, N (%)	Low education (illiterate, primary and middle school)	70 (16.7)	61 (87.1)	8 (8.8)	3 (4.3)	0.097
	High education (high school, diploma, university, and above)	384 (83.3)	262 (75.5)	59 (17.0)	27 (7.8)	
Working, N (%)	Not employed	241 (57.7)	196 (81.3)	36 (14.9)	9 (3.7)	0.005 *
	Employed	177 (42.3)	127 (71.8)	29 (16.4)	21 (11.9)	
Living Arrangements, N (%)	Alone	15 (3.6)	8 (53.3)	5 (33.3)	2 (13.3)	0.077
	With family	403 (96.4)	315 (78.2)	60 (14.9)	28 (6.9)	
Smoking, N (%)	No	389 (93.1)	311 (79.9)	49 (12.6)	29 (7.5)	<0.001 *
	Former smoker	12 (2.9)	4 (33.3)	8 (66.7)	0 (0)	
	Smoker	17 (4.1)	8 (47.1)	8 (47.1)	1 (5.9)	
Years since diagnosis, median (IQR)		11 (11–13)	13 (13–15)	8 (7–11)	6 (9–11)	<0.001 *
Other Comorbidities, N (%)		233 (55.7)	191 (82.0)	28 (12.0)	14 (6.0)	0.035 *
Number of comorbidities, median (IQR)		1 (1–2)	1 (1–2)	0 (0)	0 (0)	0.034 *
csDMARDs, N (%)		332 (79.4)	307 (92.4)	23 (6.9)	2 (0.6)	<0.001 *
bDMARDS and tsDMARDS, N (%)		132 (31.6)	82 (62.1)	31 (23.4)	19 (14.3)	<0.001 *
Corticosteroid, N (%)		137 (32.8)	123 (38.1)	8 (12.3)	6 (20)	<0.001 *
SLEDAI, N (%)	No activity (SLEDAI = 0)		197 (61.0)			
	Mild activity (SLEDAI = 1–5)		77 (23.8)			
	Moderate activity (SLEDAI = 6–10)		39 (12.1)			
	High activity (SLEDAI = 11–19)		10 (3.1)			
PsAID, N (%)	Remission <1.4			4 (6.1)		
	Low disease activity (1.4–4.1)			23 (35.3)		
	Moderate disease activity (>4.1–6.7)			22 (33.8)		
	High disease activity >6.7			16 (24.6)		
BASDAI, N (%)	<4 Inactive				8 (26.7)	
	≥4 Active disease				22 (73.3)	
Compliance, N (%)	Low	64 (15.3)	48 (75.0)	11 (17.2)	5 (7.8)	0.894
	High	354 (84.7)	275 (77.7)	54 (15.3)	25 (7.1)	
Compliance, mean (SD)		18 (2)	18 (2)	19 (2)	19 (2)	0.012 *

SLE: systemic lupus erythematosus; PsA: psoriatic arthritis; axSPA: axial spondylarthritis; SD: standard deviation; IQR: interquartile range. * Significant at a level of <0.05. csDMARDs: conventional synthetic disease-modifying antirheumatic drugs; bDMARDs: biologic disease-modifying anti rheumatic drugs; tsDMARDs: targeted synthetic disease-modifying antirheumatic drugs; SLEDAI: Systemic Lupus Erythematosus Disease Activity Index; PsAID: Psoriatic Arthritis Impact of Disease Questionnaire; BASDAI: Bath Ankylosing Spondylitis Disease Activity Index.

**Table 2:** Analysis of patient activation levels using binary logistic and linear regression.

	SLE (n = 323)		PsA (n = 65)		axSPA (n = 30)	
Odds ratio of PAM 1, (95% CI, *p*-value)	0.572	(0.572–1.568, 0.278)	2.890	(1.044–8.000, 0.041 *)	0	
Odds ratio of PAM 2, (95% CI, *p*-value)	0.809	(0.376–1.742, 0.588)	0.972	(0.415–2.278, 0.947)	0.558	(0.129–2.425, 0.437)
Odds ratio of PAM 3, (95% CI, *p*-value)	1.021	(0.637–1.636, 0.930)	1.267	(0.741–2.166, 0.387)	0.565	(0.245–1.301, 0.179)
Odds ratio of PAM 4, (95% CI, *p*-value)	1.005	(0.635–1.590, 0.983)	0.603	(0.365–1.087, 0.630)	2.460	(1.122–5.393, 0.025 *)
Coefficient of PAM 13 using linear regression (95% CI, *p*-value)	1.113	(−2.543–4.768, 0.550)	−4.272	(−8.480–0.063, 0.047 *)	5.487	(−0.426–4.768, 0.069)
Odds ratio of having high compliance	1.160	(0.625–2.154, 0.638)	0.867	(0.426–1.766, 0.695)	0.897	(0.330–2.436, 0.831)
Coefficient of ACQR using linear regression (95% CI, *p*-value)	−0.750	(−1.264–0.236, 0.004 *)	0.531	(−0.067–1.129, 0.082)	0.930	(0.093–1.768, 0.030 *)

SLE: systemic lupus erythematosus; PsA: psoriatic arthritis; axSPA: axial spondylarthritis; PAM: patient activation measure; CI: confidence intervals. * Significant at a level of *p* < 0.05. PAM 1: disengaged and overwhelmed; PAM 2: becoming aware but still struggling; PAM 3: taking action and gaining control; PAM 4: maintaining behaviors and pushing further.

## Data Availability

Data is available on request from the corresponding author.

## Supplementary Materials

The following supporting information can be downloaded at: https://www.mdpi.com/article/10.3390/healthcare13010071/s1, Figure S1: correlation between total patient activation measure (PAM) 13 score and PsAID score PsAID: Psoriatic Arthritis Impact of Disease; Figure S2: correlation between total patient activation measure (PAM) 13 score and BASDAI score, BASDFI: Bath Ankylosing Spondylitis Disease Activity Index. Figure S3: PAM level of the entire population.
