# Supplementary material for: Exploring Patient Activation and Compliance in Patients with Different Rheumatological Disorders
Source: Healthcare (Basel). 2025 Jan 2;13(1):71. doi: 10.3390/healthcare13010071 (PMC11720284; doi:10.3390/healthcare13010071)
Supplement: Supplementary file 1 [file healthcare-13-00071-s001.zip › healthcare-3376895-supplementary.pdf]

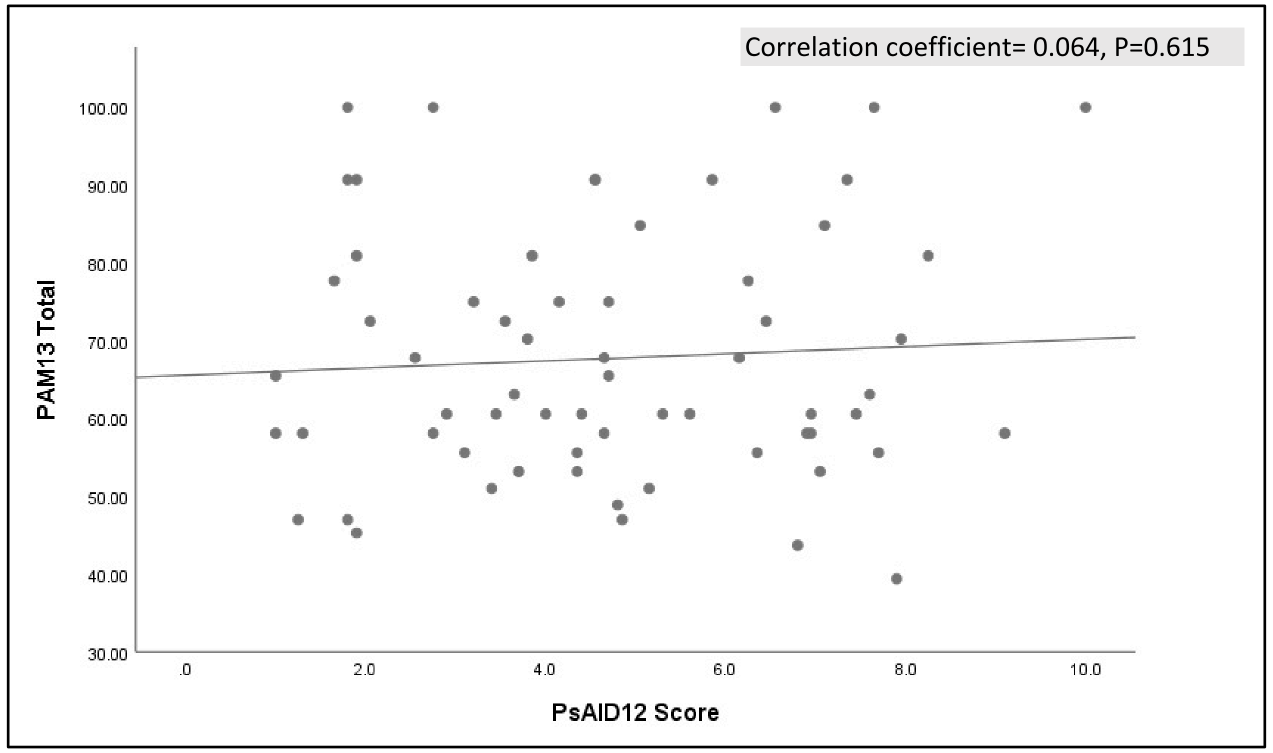

Figure S1 correlation between total patient activation measure (PAM) 13 score and PsAID score
PsAID: Psoriatic Arthritis Impact of Disease

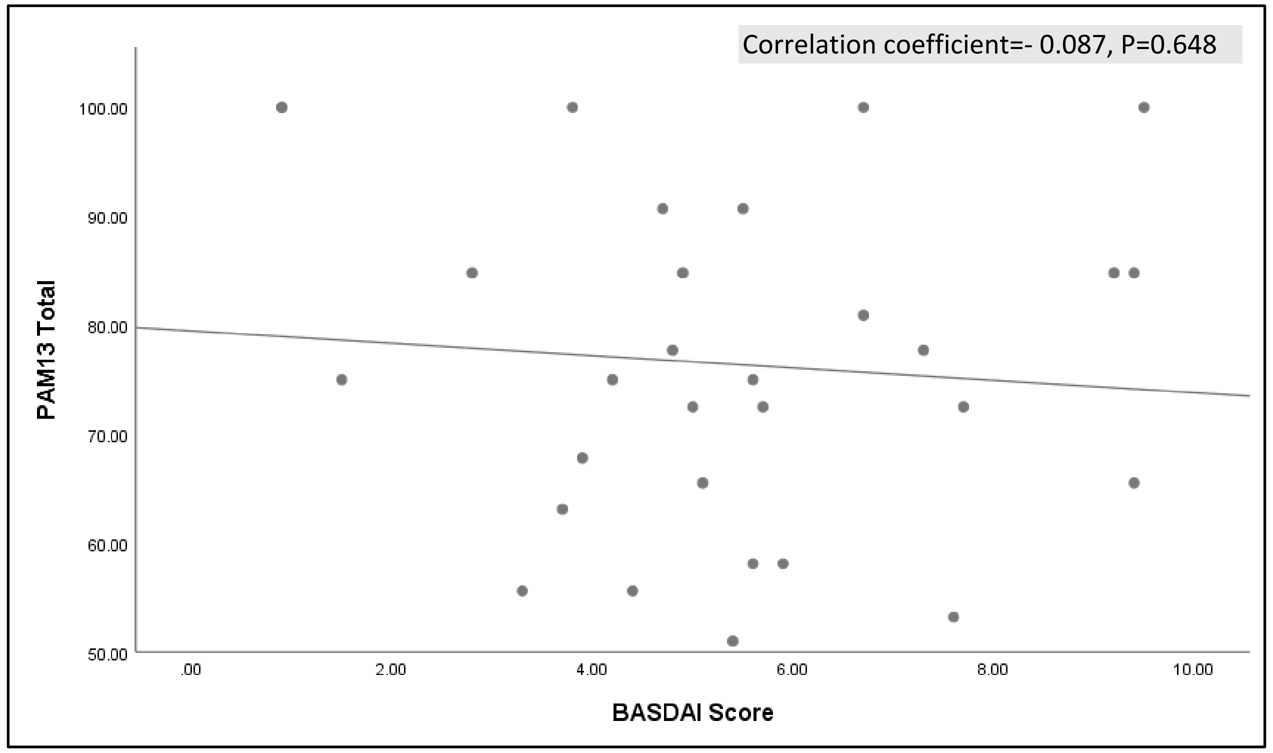

Figure S2 correlation between total patient activation measure (PAM) 13 score and BASDAI score
BASDFI: Bath Ankylosing Spondylitis Disease Activity Index

Supplementary

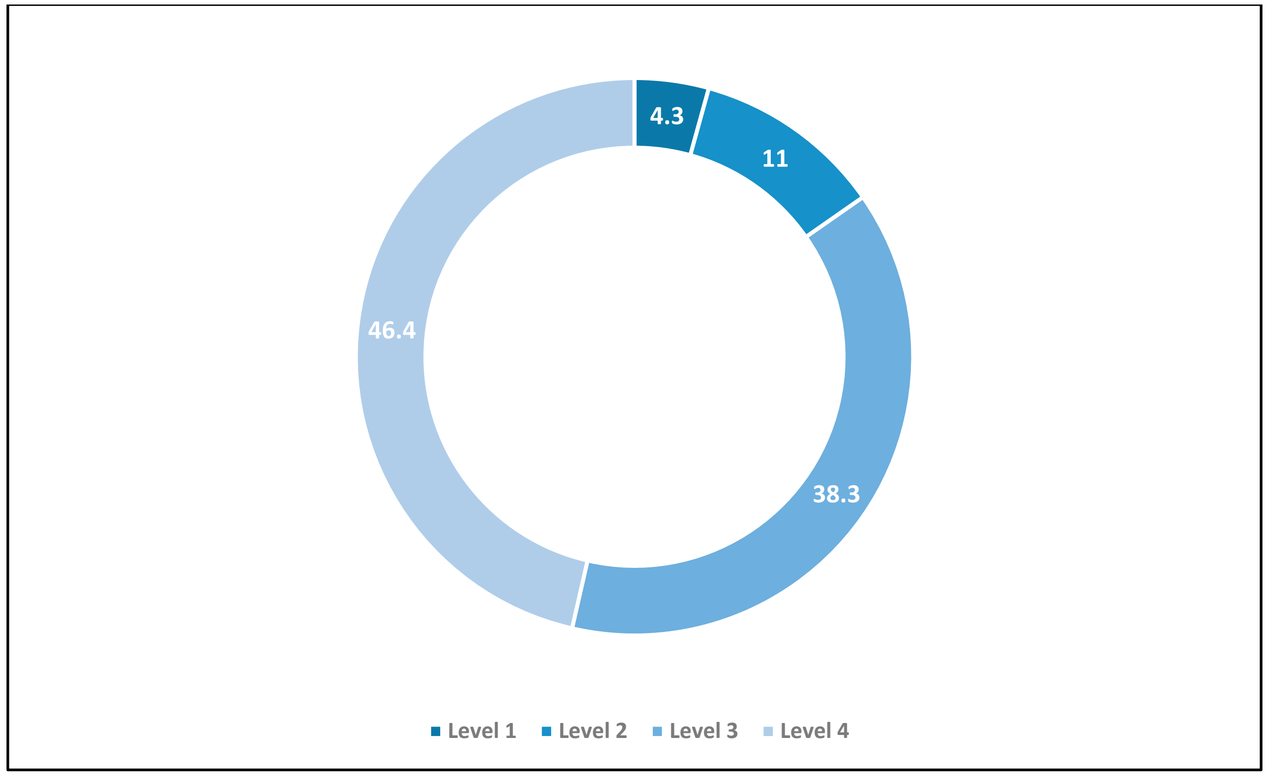

Supplementary Figure S3 PAM level of the entire population
